# Association Between MitoScore, BMI, and Body Fat Percentage as a Predictive Marker for the Outcome of In-Vitro Fertilization (IVF)

**DOI:** 10.7759/cureus.27367

**Published:** 2022-07-27

**Authors:** Himanshu Arora, Ineabelle Collazo, Juergen Eisermann, Nicholas Hendon, Manish Kuchakulla, Kajal Khodamoradi, Joginder Bidhan, Alexandra Dullea, Isaac Zucker, Zahra Khosravizadeh, Parth Shah, Maria Bustillo

**Affiliations:** 1 Urology, University of Miami, Miami, USA; 2 South Florida Institute for Reproductive Medicine, IVFMD, Miami, USA; 3 Obstetrics and Gynecology, University of Miami, Miami, USA; 4 Urology, Weill Cornell Medical Center, New York, USA; 5 Urology, Florida International University, Miami, USA; 6 Anatomy, Tehran University of Medical Sciences, Tehran, IRN; 7 Medicine, American University of Antigua, Osbourn, ATG

**Keywords:** body mass index, assisted reproductive technique, body fat, fertility, mitoscore

## Abstract

Background

Infertility is defined as the inability to establish a pregnancy within 12 months of regular and unprotected sexual intercourse. In response to these problems, assisted reproductive techniques (ARTs) have made profound impacts on the therapeutic management of infertility. However, in-vitro fertilization (IVF) success rates are confounded by several internal and external factors. A relatively new approach to embryo assessment is known as MitoScore (Igenomix, Miami, USA). As a result, we sough to evaluate whether MitoScore can help in predicting in IVF outcomes, and to assess the relationship between MitoScore, BMI, and body fat percentage in determining the success of ARTs.

Methods

Using retrospective cohort, a study population consisting of 166 women aged 26-43 who were undergoing ART with pre-implantation genetic testing for aneuploidy (PGT-A) was assessed to determine if MitoScore, BMI, and body fat percentage impacted IVF outcomes.

Results

MitoScore, BMI, and body fat percentage were significantly lower in pregnant women as compared to non-pregnant women. Furthermore, MitoScore was correlated with subclasses of IVF outcomes (delivery, biochemical pregnancy, and spontaneous abortion) and was found to be positively correlated with BMI in patients with biochemical pregnancies.

Conclusion

Our findings suggest that MitoScore, BMI, and body fat percentage could act as critical parameters in determining the success of ART. However, the association between MitoScore, BMI, and body fat percentage does not appear to be a significant confounding factor to determine pregnancy outcome at this stage. Still, many factors need to be considered to establish the correlation reliably.

## Introduction

Infertility is defined as the inability to establish a pregnancy within 12 months of regular and unprotected sexual intercourse [1]. Current studies suggest that approximately 8-12% of the global population is affected by infertility [2]. In response to these problems, assisted reproductive techniques (ARTs) have made profound impacts on the therapeutic management of infertility. However, the in-vitro fertilization (IVF) success rates are confounded by several internal and external factors. The predictive factors for successful pregnancy after ARTs have long been studied [3]. Among those studied, epidemiological factors such as lifestyle, family history, and overall body attributes are considered to modulate the endocrine system and affect IVF outcomes [4-6]. It has been demonstrated that being overweight or having a higher BMI are associated with unsuccessful IVF [7]. Also, overweight women with higher levels of fatty acids and fat hormones have decreased fecundity and impaired IVF outcomes [8-10]. Factors affecting ovarian function such as female age, number of oocytes, duration of subfertility, and basal follicle-stimulating hormone (FSH) have been identified as predictors for success in IVF [11]. Furthermore, some factors including a previous pregnancy, the diagnosis after fertility workup, and the number of previous failed IVF attempts are probable predictors of IVF success [11]. However, even after considering these aspects, IVF success rates have remained low [12,13].

A relatively new approach to embryo assessment is known as MitoScore. MitoScore is a value that represents the normalized mitochondrial DNA (mtDNA) quantity in embryos [14]. Mitochondria are involved in the regulation of many cellular processes, such as apoptosis, amino acid synthesis, and generation of ATP. Therefore, the quantity of mtDNA has been related to the energy supply of the embryo [13]. However, an increased amount of mtDNA is related to poor implantation potential and reduced metabolic fuel for oocyte maturation [15]. It has been estimated that mature oocytes have more than 150,000 copies of mtDNA. Although mature oocytes with less than 4,000 mtDNA copies can be fertilized and normally develop to blastocyst stage, the threshold of 40,000-50,000 mtDNA copies is needed for post-implantation development of mature oocyte [16]. The majority of embryos at cleavage stage with low mtDNA copy numbers are unable to complete post-implantation development [16]. A negative correlation has been reported between maternal age and number of mtDNA copies in human oocytes [17], and poor oocyte quality in ovarian insufficiency has also been correlated with low MitoScore [18]. Mitoscore, thus, could offer invaluable insights into IVF outcomes [15,19]. It has been suggested that MitoScore is a potential novel biomarker for IVF treatment, revealing the incapability of a blastocyst with normal chromosomes to produce a viable pregnancy [20]. However, there are contradictory results about the validity of MitoScore as a predictor of embryo implantation [20].

Understanding the factors affecting IVF outcome may be useful for predicting results and lead to the development of effective strategies to improve the success rate of IVF treatment [21]. To answer the question of whether or not MitoScore can help in predicting outcomes of IVF, as well as to determine the relationship between MitoScore, BMI, and body fat percentage in determining the success of ARTs, we performed a prospective cohort study using data from women undergoing ARTs with pre-implantation genetic testing.

## Materials and methods

Study population

The study population consisted of 166 women, 26-43 years of age (mean of 36.08 years), and mean BMI of 24.99, who were undergoing ART with pre-implantation genetic testing for aneuploidy (PGT-A). Of those 166 women, 36 women did not conceive and 130 became pregnant, with 104 having delivery. Furthermore, 15 biochemical pregnancies and 11 spontaneous abortions (SAb) were reported. PGT-A and MitoScore testing (on euploid embryos) were performed by Igenomix, Miami, USA, for all 130 pregnant women.

The protocol of this retrospective study was approved by our institutional review board, the ethics committee of Clinical Research ART data gathering complies with US law on assisted reproductive technologies (The Fertility Clinic Success Rate Act (Wyden bill) of 1992).

Oocyte retrieval, embryo biopsy, and culture conditions

Patients underwent controlled ovarian stimulation using standardized protocols. When at least two follicles had reached ≥ 18 mm in diameter, human chorionic gonadotropin (hCG) (Pregnyl (MSD, USA) at either 5.000 or 10.000 international units (IU) was administered to trigger oocyte maturation. Oocyte retrieval was performed 36 hours after hCG administration under transvaginal ultrasonography guidance. After oocyte denudation, intracytoplasmic sperm injection (ICSI) was performed and fertilization was assessed 17-20 hours after microinjection and confirmed by the presence of two pronuclei and two polar bodies. The embryos were washed and cultured in cleavage media G-1 PLUS (Vitrolife, USA) for two to three days. After assessment on day three, the individual embryos were placed in G-2™ PLUS and OVOIL™ (Vitrolife, USA) in an atmosphere containing 5% O_2_ and 5.5% CO_2_ at 37 °C.

Embryo biopsy was performed on day five at the blastocyst stage in all cases, and a single blastomere was withdrawn from each embryo with laser-assistance [22,23]. The most common PGT-A indications in this study were patients with a history of recurrent miscarriage, repetitive implantation failure, advanced maternal age, or male factor infertility. For PGT, we used amplified DNA from euploid embryos from the aforementioned patients, who then underwent elective single embryo transfer.

Next-generation sequencing (NGS)

A NGS platform (ReproSeq, Thermo Fisher Scientific) was used to analyze the biopsy samples. After using a whole-genome amplification (WGA) protocol for all individual samples, the library preparation consisted of incorporating individual barcodes for the amplified DNA of each embryo. Following isothermal amplification and enrichment, sequencing was performed in a 316- or a 318-chip using a Personal Genome Machine (PGM) sequencer (Thermo Fisher Scientific). Ion Reporter software (Thermo Fisher Scientific) was used for the sequencing analysis and data interpretation. The embryos were diagnosed as chromosomally normal, abnormal, or chaotic abnormal. The chaotic embryos were defined as those that displayed a complex pattern of aneuploidies involving more than six chromosomes. The mtDNA copy number was detected from the same sample in Igenomix. For the MitoScore calculations, an optimized algorithm was applied that used the output dataset obtained from the PGT analysis, which was comprised of a mixture of mtDNA reads and nuclear DNA (nDNA) reads. To calculate the relative mtDNA copy number score in embryos, the number of reads after filtering were mapped to the mitochondrial genome and were divided by the number of reads that mapped to the nuclear genome. This allowed the normalization of each batch, thus reducing variability during NGS experiments (e.g., independent calculations of the number of cells obtained in each biopsy).

Statistical analysis

GraphPad Prism (GraphPad Software) was used for statistical analysis. All variables were presented as the means ± standard error of the mean (SEM). The statistical significance between two groups was estimated by unpaired two-tailed t-test. Multiple group comparisons were performed using a one-way analysis of variance with least significant difference test. In all cases, p < 0.05 was considered statistically significant.

Patient data was classified into negative and positive pregnancy outcomes. In addition, positive pregnancy outcomes were subclassified into delivery, biochemical pregnancy, and SAb.

## Results

MitoScore, BMI, and body fat percentage are significantly correlated with IVF outcomes

**Figure 1:**
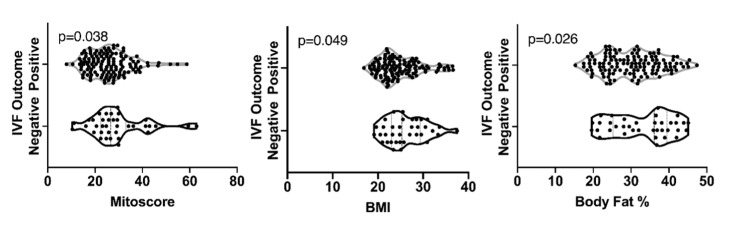
Comparison between positive (top) and negative (bottom) IVF outcomes dependent on Mitoscore, BMI, and body fat percentage IVF: In-vitro fertilization P values are noted at the top of each graph.

The results from MitoScore, BMI, and body fat percentage in the population study with respect to IVF outcomes indicated that in general BMI, body fat percentage and MitoScore were significantly lower in patients who had a positive pregnancy test as compared to women with negative pregnancy tests (MitoScore: p = 0.0381, BMI: p = 0.0494, body fat percentage: p = 0.0265) (Table 1) (Figure 1). The mean values of MitoScore, BMI, and body fat percentage for patients achieving a positive pregnancy test were 25.92 ± 8.905, 24.7 ± 4.495, and 30.21 ± 7.407, respectively (Table 1). These findings support the evidence that these variables are inversely correlated with IVF outcomes.

**Table 1:** Characteristics of patient study population with respect to MitoScore (1A), BMI (1B), body fat percentage (1C), and correlation between MitoScore with BMI and body fat percentage (1D), respectively SAb: Spontaneous abortions; SEM: Standard error of the mean; BIOCHEM: Biochemical pregnancy * indicates significance with p  <0.05. All p values were calculated against Not Pregnant Outcome

Table 1A				
Pregnancy Outcome	No of Patients	%	MitoScore±SEM	p-value
Not Pregnant	36	21.7	29.11±11.51	ref
Pregnant	130	78.3	25.92±8.905	0.0381*
SAb	11	6.63	26.14±9.076	0.3252
BIOCHEM	15	9.04	27.36±9.647	0.1893
DELIVERED	104	62.7	25.74±8.879	0.0362*
Total	166	100		
Table 1B				
Pregnancy Outcome	No of Patients	%	BMI±​​​​​​​SEM	p-value
Not Pregnant	36	21.7	26.09±4.366	ref
Pregnant	130	78.3	24.7±4.495	0.0494*
SAb	11	6.63	23.75±5.349	0.2462
BIOCHEM	15	9.04	24.98±5.531	0.0546
DELIVERED	104	62.7	24.8±4.278	0.0613
Total	166	100		
Table 1C				
Pregnancy Outcome	No of Patients	%	%Body Fat±​​​​​​​SEM	p-value
Not Pregnant	36	21.7	32.98±7.999	ref
Pregnant	130	78.3	30.21±7.407	0.0265*
SAb	11	6.63	28.38±7.536	0.4019
BIOCHEM	15	9.04	32.31±7.038	0.0315*
DELIVERED	104	62.7	30.26±7.431	0.0326*
Total	166	100		
Table 1D				
Pregnancy Outcome	No of Patients	%	MitoScore-BMI (p value)	MitoScore- Body Fat % (p value)
Not Pregnant	36	21.7	ref	ref
SAb	11	6.63	0.6295	0.971
BIOCHEM	15	9.04	0.0369*	0.0691
DELIVERED	104	62.7	0.4834	0.6323

Correlation of MitoScore, BMI, and body fat percentage with subclasses of IVF outcomes

**Figure 2:**
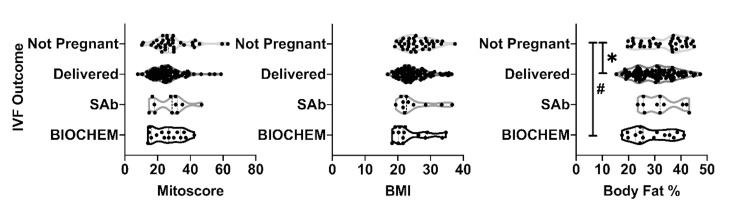
Comparison between IVF outcomes (non-pregnant, delivered, spontaneous abortion, and biochemical pregnancy) by Mitoscore, BMI, and body fat percentage IVF: In-vitro fertilization; SAb: Spontaneous abortions; BIOCHEM: Biochemical pregnancy Mean body fat percentage was significant (p < 0.05) between non-pregnant and biochemical pregnancy, as well as non-pregnant and delivered.

Among MitoScore, BMI, and body fat percentage, MitoScore appears to be a more potent confounding factor with respect to its inverse correlation with IVF outcomes (Figure 1). Correlation between IVF outcomes (negative pregnancy and subclasses of positive pregnancy) and MitoScore, BMI, or body fat percentage showed that MitoScore was significantly and positively correlated with BMI in patients with biochemical pregnancy (p = 0.0369); however, it was not correlated in patients with delivery (p = 0.629) or those with SAb (p = 0.483) (Table 1D). On the other hand, MitoScore was not found to be significantly correlated with body fat percentage in any of the subclasses of positive pregnancy (Figure 2).

Additionally, we found a significant difference in the mean of body fat percentage between negative pregnancy and ongoing pregnancy and between negative pregnancy and biochemical pregnancy (p < 0.05) (Figure 2). Finally, these results suggest that there is a correlation between MitoScore and BMI with respect to IVF outcomes, and that it is important to consider subclasses of IVF outcomes while determining the effects of each of these confounding factors (MitoScore, BMI, and body fat percentage).

## Discussion

Our findings suggest that MitoScore, BMI, and body fat percentage could act as critical parameters in determining the success of ART. Among MitoScore, BMI, and body fat percentage, MitoScore appears to be a more potent confounding factor with respect to its a) inverse correlation with IVF outcome, b) significant correlation with BMI in one of the subclasses of IVF outcome (biochemical pregnancy), and c) significantly difference when comparing a negative pregnancy test and subclasses of IVF outcome.

IVF was first successful in 1978 and, since then, over 5 million children have been conceived using this technology. Recently, approximately 1.6% births in the United States resulted from IVF [24]. The growing use of IVF presents significant potential and realized costs as well as burdens to families [25]. Furthermore, short term psychological adjustment within unsuccessful cycles is an important health consequence to consider [26]. For both the cost and the psychological effects, it is important to find new ways to determine beforehand if an IVF cycle will be successful. Although there are many variables associated with IVF and its success, the majority of independent studies have focused on studying them separately, which itself could act as a limiting factor. The ability to have multiple factors that can predict IVF outcomes and understanding their associations with each other can lead to improving the health of patients prior to ARTs in order to reduce potential burdens and negative connotations with this process. 

Recently, it has been suggested that the assessment of MitoScore can be a novel way to identify embryos with the highest reproductive capacity to result in live births [14]. According to initial studies, increased MitoScore is correlated with aneuploidy and reduced implantation potential [15,19]. Boynukalin et al. reported that live birth rate was significantly higher among cases with a lower MitoScore following frozen single euploid blastocyst transfer [3]. In agreement with our findings, Fragouli et al. and Diez-Juan et al. have both shown evidence that MitoScore is negatively correlated with IVF outcomes [15,19]. Both studies associated higher MitoScore levels with poorer implantation potential and reduced metabolic fuel for oocyte maturation.

Embryos with lower implantation potential and lower trophectoderm quality have a higher MitoScore, probably because of increased mitochondrial biogenesis. According to the association between trophectoderm quality and MitoScore, it seems that changes in mitochondrial biogenesis have a negative effect on the proliferative capacity of trophoblast and subsequent implantation [27]. Although stress results in elevating the metabolic output of the embryo as a compensatory mechanism, there is no document of association between perceived stress and increased MitoScore [20]. However, there is a hypothesis that MitoScore can be considered as an indicator of energetic stress in an embryo and therefore can be used for predicting implantation capacity [15].

In Fragouli et al.’s study, the aneuploidy embryos showed an elevated MitoScore [19], whereas Bayram et al.’s study showed a higher MitoScore in euploid embryos [14]. According to these contrary results, there is not enough information about the direct association between embryonic aneuploidy and elevated MitoScore [14].

Collazo et al. demonstrated that although MitoScore is different between embryo qualities, several factors must be considered to determine the importance of MitoScore as a predictor for ART outcome [28]. Conversely, there have been several other studies which contradict these findings and reveal no significant differences, making further exploration of embryo qualities necessary [29-31]. Klimczak et al. reported that although embryos with a higher MitoScore showed poorer quality, in an analysis of only euploid embryos, MitoScore does not appear to be related to embryo quality. Also, there is no significant association between MitoScore and implantation or ongoing pregnancy [31]. Treff et al. and Victor et al. have both reported that the difference in MitoScore between implanted blastocysts and non-implanted blastocysts is insignificant, and also that no association between MitoScore and blastocyst viability is shown [29,30]. It should be mentioned that these contradictory results may be due to the use of different methods for evaluating MitoScore. It appears that identification of the best methods for evaluating MitoScore and the factors that lead to a decreased MitoScore in an embryo at the blastocyst stage are essential [3].

While MitoScore is relatively new and controversial, studies analyzing BMI and body fat percentage on IVF outcomes have been long-established [4,7,8,32]. To support our data, a systematic review and meta-analysis showed that BMI is linked to a lower live-birth rate in women undergoing IVF/ICSI [33]. Furthermore, a retrospective cohort study from 2009 to 2015 by Kudesia et al. analyzing 51,198 patients studied the impact of BMI on IVF outcomes. Results demonstrated that being overweight or having a higher BMI was associated with failed outcomes [7]. Overweight women with higher levels of fatty acids and fat hormones had decreased fecundity and impaired IVF outcomes [8-10].

Thus, the data behind MitoScore, BMI, and body fat percentage is strong at the moment. However, there is no current analysis studying the correlation between these three variables. Our study is among the first to analyze the association between MitoScore, BMI, and body fat percentage with respect to IVF outcomes.

Our findings suggest that MitoScore, BMI, and body fat percentage could act as critical parameters in determining the success of ART. However, the association between MitoScore, BMI, and body fat percentage does not appear to be a significant confounding factor to determine pregnancy outcome at this stage. Still, many factors need to be considered to establish the correlation reliably. Further analyses of other determinants such as age, infertility diagnosis, and inflammatory markers are underway to establish the significance of MitoScore. 

Our study has both strengths and limitations. In the current study, we utilized results from 166 women (130 pregnant and 36 nonpregnant) to determine the role of MitoScore, BMI, and body fat percentage as confounding factors in IVF outcome. The results suggest that MitoScore, BMI, and body fat percentage are useful considerations in predicting the success of ART. However, their association with each other requires stratifications, which include subclasses of IVF outcomes, patient age, thyroid-stimulating hormone (TSH), anti-Müllerian hormone (AMH), etc. Our findings contribute original evidence to the font of knowledge in the effort to improve IVF outcomes. While our findings have durability, there are also some considerations that may impact the power of these observations. One of the limitations is that age was not considered as a variable in our analysis, as it has been reported that MitoScore is altered in female patients with progressing age [19]. While this would not alter the results, it might provide some possible explanation to the outcomes we observed. Another limitation is our focus on IVF outcome, which is only one measure, since there may be many other factors that contribute to pregnancy viability.

## Conclusions

At this time, the use of MitoScore is still controversial as not all studies have been able to find a clear link between increased MitoScore and IVF outcomes. Our study tried to answer these discrepancies by utilizing multiple variables (such as BMI and body fat percentage). However, having made some interesting observations, it is critical to include more aspects which include, but are not limited to, patient age, TSH, AMH, and thyroid antibodies to help predict ART outcomes significantly.
